# Impact of social vulnerability on glycemic control and diabetes technology use in youth with type 1 diabetes

**DOI:** 10.1186/s12902-025-02161-2

**Published:** 2026-01-24

**Authors:** Nicholas R. Banull, Melanie Bryan, Stephen I. Stone, Hope Shimony, Amanda Ingram, Ana María Arbeláez

**Affiliations:** Division of Pediatric Endocrinology, Washington University School of Medicine in St. Louis, Campus Box 8116, 660 South Euclid, St. Louis, Missouri 63110 USA

**Keywords:** Diabetes, Continuous glucose monitoring, Insulin pump, Social vulnerability, Glycemic control, Diabetic ketoacidosis

## Abstract

**Background:**

Diabetes technology has been shown to improve glycemic control in people with type 1 diabetes (T1DM). Despite their benefits, these technologies are used less frequently by youth from lower socioeconomic backgrounds and racial minorities worldwide. This study aimed to examine the relationship between social vulnerability, disparities in diabetes technology use, and glycemic control in youth with T1DM.

**Methods:**

This retrospective billing code analysis included 1,460 patients with T1DM, aged ≤ 23 years old, who were seen at a large academic diabetes center in the Midwest between January 2020 and April 2022, before the expansion of insurance coverage for diabetes technology in Missouri. We analyzed data on number of visit encounters, use of a continuous glucose monitor (CGM), use of an insulin pump, area of deprivation index (ADI) score, age, sex, race, and type of insurance. A predictive multiple linear regression model was used to assess mean hemoglobin A1c (HbA1c). A negative binomial regression was used to assess diabetic ketoacidosis (DKA) occurrences.

**Results:**

There was statistically significant lower usage of diabetes technology in Black patients, those on Medicaid, and those with higher social vulnerability (*p* < 0.001). Increased rates of DKA were associated with older age, higher HbA1c, Black racial identity, and insulin pump use. Conversely, independent insulin pump or CGM use was significantly associated with lower HbA1c levels, especially among patients from the most disadvantaged groups (*p* < 0.001).

**Conclusions:**

This study highlights that independent insulin pump or CGM use can mitigate the negative impacts of social vulnerability and race on long term glycemic control in youth with type 1 diabetes, emphasizing the need for greater access to these essential technologies.

## Background

Advancements in diabetes technology, particularly continuous glucose monitors (CGMs) and continuous subcutaneous insulin infusion (CSII) via insulin pumps, have been shown to improve glycemic control in youth with type 1 diabetes mellitus (T1DM) [1, 2] when compared with multiple daily injections [3]. These devices have been shown to increase time in the target glucose range, lower rates of hypoglycemia, and reduce admissions for diabetic ketoacidosis (DKA) [3–5].

Despite these significant benefits, the use of CGMs, insulin pumps, and closed-loop insulin delivery systems in children worldwide remains low due to cost and barriers to adopting this technology. Although the uptake of these devices is increasing in the US, their usage is notably less frequent among children from lower socioeconomic backgrounds and among ethnic and racial minorities [6–14]. This disparity in the use of diabetes technology may contribute to the well-documented differences in clinical outcomes for Hispanic and non-Hispanic Black individuals with T1DM as compared to their White peers [15]. Furthermore, research indicates that racial disparities in diabetes outcomes persist even after controlling for socioeconomic status, suggesting that mistrust, implicit bias, and systemic racism may have an impact on diabetes outcomes for non-White individuals with T1DM [16, 17].

This study aimed to assess the relationship between social vulnerability, disparities in diabetes technology use, and clinical outcomes in youth with Type 1 diabetes (T1DM). Understanding the mechanisms that drive these disparate outcomes is crucial to providing more equitable, comprehensive, and appropriate care to all children with T1DM and their families.

## Methods

### Participants and data collection

A retrospective data analysis was conducted using billing data from patients with T1DM, who were under 23 years old, and had been seen at Washington University Pediatric Diabetes Clinics between January 2020 and April 2022. Patients with an E10 International Statistical Classification of Diseases and Related Health Problems, Tenth Revision (ICD-10) diagnosis, corresponding to T1DM, were included in the study. Although this time frame encompasses the COVID-19 pandemic, patient care for those with T1DM was not interrupted at this academic center during the study period. However, some visits were switched to virtual appointments from in-office visits from March 2020 to July 2020. This change was made to ensure that patients met the annual visit requirements set by the American Diabetes Association guidelines and to prevent any interruption in their care.

### Outcome variables

The primary outcome variables were glycated hemoglobin (HbA1c), which reflects chronic glycemic control, and rates of diabetic ketoacidosis (DKA). Each encounter included in this study corresponded to a HbA1c measurement. Mean HbA1c was estimated by calculating the average of all HbA1c measurements for each individual patient over the two-year study period including HbA1c measures at diagnosis. DKA occurrences were determined by the number of inpatient admissions in DKA for each patient over the two-year study period. Current Procedural Terminology (CPT) codes and demographic variables were extracted from the electronic medical health record (EMHR) to assess potential covariates that could have influenced clinical outcomes. These covariates included age, sex, race, social disparity, insurance, number of visit encounters, use of an insulin pump, and/or a continuous glucose monitor. Individuals were considered pump or CGM users if they had utilized the respective technology for more than one month at any point during the study period. Throughout the study, all patients in the practice using CGM used real-time continuous glucose monitors (rtCGM). Social disparity was assessed using the Area Deprivation Index (ADI), which provides an objective comparison of relative socioeconomic conditions of neighborhoods across the United States, based on census data from the 2018 American Community Survey. The ADI takes into account demographic and socioeconomic factors that affect a community’s ability to respond to external stressors, such as neighborhood household income, housing quality, education, and employment. The ADI scores were calculated using patients’ home addresses and The Neighborhood Atlas® software from the University of Wisconsin [18]. Higher ADI scores indicate a higher level of social deprivation.

### Statistics

Descriptive statistics were calculated for all variables, including mean, standard deviation, and median. Univariate analysis was performed to identify potential influencing factors on HbA1c. Variables were included in a predictive multiple linear regression model based on either statistical significance or clinical relevance. To assess for normality, distribution of unstandardized residuals and normalized Q-Q plots were analyzed. Multicollinearity was assessed by calculating variance inflation factors using a linear regression model. Poisson regression was initially performed on non-normally distributed outcome variables to assess for overdispersion. Negative binomial regression with a log link was performed using DKA as an output variable and estimated using fisher scoring with maximum likelihood and type III Wald Chi-Square tests. Results were considered statistically significant if *p* < 0.05 in two-tailed tests. Analyses were conducted using the statistical package SPSS© Version 27. Graphics were produced using Adobe Illustrator, SPSS, and Vega-Altair [19]. This study was approved by Washington University’s Human Research Protection Office Committee and waiver of consent was granted.

## Results

**Fig. 1:**
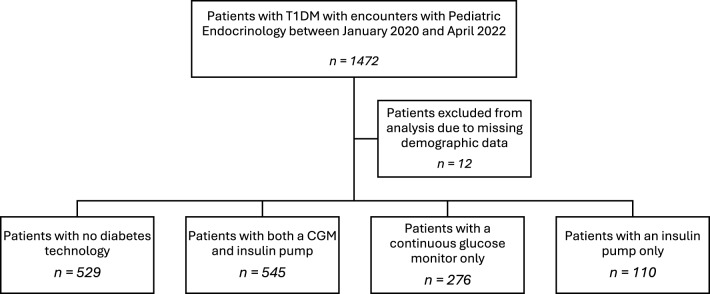
Total number of patients included in the study after excluding for missing data. The distribution of patients using different diabetes technologies is also shown

Between January 2020 and April 2022, a total of 1,472 unique patients with T1DM were seen at the Department of Pediatric Endocrinology Diabetes outpatient clinics at Washington University in St. Louis (see Fig. 1). Twelve patients were excluded from the analysis due to missing demographic data or an inability to calculate ADI, for a total of 1,460 patients included in this study. These patients had 7,410 encounters during the analysis period.

### Descriptive statistics

Population characteristics are summarized in Table 1. The mean age was 13.27 (SD = 4.4) years, and 54.6% of the patients being male. The patients’ racial composition was 84.6% White, 13.2% Black, and 2.2% other. 66.4% of patients were on managed care insurance, 26.5% were on Medicaid, and 7.1% were on other types of insurance. The overall ADI score for the cohort was 57.0 (SD = 24.7.) The mean ADI of Black patients was 73.80 (SD = 23.6), while white patients had a mean ADI of 54.38 (SD = 23.9). 37.3% of patients used pump and CGM, 7.5% used pump only, 18.9% used CGM only, and 36.2% did not use diabetes technology.

**Table 1:** Characteristics of study participants

	**No Technology** (*n* = 529)	**CGM Only** (*n* = 276)	**Pump Only** (*n* = 110)	**Pump and CGM** (*n* = 545)	**Total** (*n* = 1460)
**Age**					
0–1 y	2 (66.7)	1 (33.3)	0	0	3
1–5 y	32 (36.8)	22 (25.3)	4 (4.6)	29 (33.3)	87
5–11 y	144 (36.4)	60 (15.2)	34 (8.6)	157 (39.8)	395
11–18 y	304 (35.6)	165 (19.3)	67 (7.8)	318 (37.2)	854
18–25 y	47 (38.8)	28 (23.1)	5 (4.1)	41 (33.9)	121
Mean Age (σ)	13.3 (3.3)	13.5 (4.6)	13.4 (4.1)	13.2 (4.2)	13.3 (4.4)
**Sex**					
Male	307 (38.5)	156 (19.6)	52 (6.5)	282 (35.3)	797
Female	222 (33.5)	120 (18.1)	58 (8.7)	263 (39.7)	663
**Insurance**					
Commercial	1 (16.7)	0	0	5 (83.3)	6
Managed Care	270 (27.8)	192 (19.8)	67 (6.9)	440 (45.4)	969
Managed Medicaid	216 (55.8)	66 (17.1)	34 (8.8)	71 (18.3)	387
Medicaid	21 (60)	6 (17.1)	3 (8.6)	5 (14.3)	35
Medicare	1 (100)	0	0	0	1
No Coverage	2 (40)	1 (20)	2 (40)	0	5
Other	13 (26.0)	10 (20)	3 (6.0)	24 (48.0)	50
Self-Pay	5 (71.4)	1 (14.3)	1 (14.3)	0	7
**Race**					
American Indian or Alaska Native	2 (50)	0	1 (25)	1 (25)	4
Asian	6 (35.3)	3 (17.6)	2 (11.8)	6 (35.3)	17
Black or African American	116 (60.1)	41 (21.2)	11 (5.7)	25 (13.0)	193
Other Pacific Islander	3 (33.3)	3 (33.3)	0	3 (33.3)	9
Other	2 (50)	1 (25)	0	1 (25)	4
White	400 (32.4)	228 (18.5)	96 (7.8)	509 (41.2)	1233
**Mean HbA1c % (σ)**	9.4 (2.2)	8.8 (1.9)	8.7 (1.4)	8.1 (1.3)	8.7 (1.9)
**Mean ADI (σ)**	64.0 (24.3)	55.0 (23.7)	59.4 (23.33)	50.3 (23.9)	56.8 (24.7)
**Mean Number of DKA Admissions (σ)**	0.77 (2.0)	0.82 (1.7)	0.83 (1.5)	0.53 (1.6)	0.69 (1.8)
**Encounters**					
Total Encounters	2028 (27.4)	1650 (22.3)	564 (7.6)	3168 (42.8)	7478
Mean Encounters (σ)	3.8 (2.8)	6.0 (2.8)	5.1 (4.8)	5.8 (2.2)	5.1 (3.0)

The mean number of encounters per patient was 5.1 over the study period (SD = 3.0). Each patient encounter was associated with a corresponding HbA1c result. The overall mean HbA1c was 8.72% (SD = 1.9) for all patient encounters. For White patients, the mean HbA1c was 8.5% (SD = 1.7); while Black patients had a mean HbA1c of 10.5% (SD = 2.3). Patients without diabetes technology had a mean HbA1c of 9.4% (SD = 2.2). The mean HbA1c for patients using a CGM was 8.8% (SD = 1.9), for those using an insulin pump was 8.7% (SD = 1.4), and patients using both a CGM and an insulin pump had a mean HbA1c of 8.1% (SD = 1.3)

The mean number of DKA admissions per patient was 0.69 (SD = 1.8). White patients had a mean of 0.52 (SD = 1.4) DKA admissions, while Black patients averaged 1.79 (SD = 3.1) DKA admissions. For patients without diabetes technology, the mean number of DKA admissions was 0.77 (SD = 2.0), for those using an insulin pump it was 0.83 (SD = 1.6), and for those using a CGM it was 0.82 (SD = 1.7). Patients utilizing both a CGM and an insulin pump had a mean number of 0.53 (SD = 1.6) DKA admissions.

### Multivariable regression analysis

#### HbA1c

**Fig. 2:**
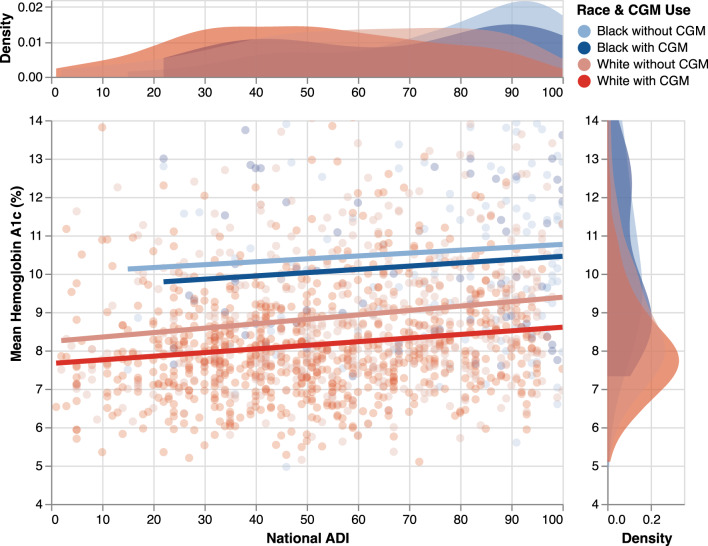
Linear regression plot of mean HbA1c (%) by national area deprivation Index and stratified by patients’ CGM usage and race. The kernel density plot (distribution of observations) of HbA1c is shown to the right, and the kernel density plot of the ADI scores is shown above

**Fig. 3:**
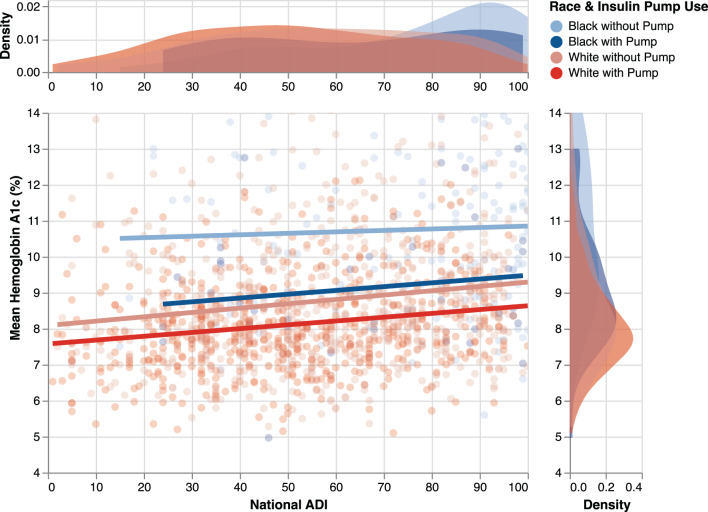
Linear regression plot of mean HbA1c (%) by national area deprivation Index and stratified by patients’ insulin pump usage and race. The kernel density plot (distribution of observations) of HbA1c is shown to the right, and the kernel density plot of the ADI scores is shown above

Unstandardized residuals did not demonstrate a normal distribution for HbA1c (Kolmogorov-Smirnov *p* < 0.001). Given large sample size, multiple linear regression was performed using HbA1c as an outcome variable. Univariate linear regression analysis showed that ADI, insulin pump use, CGM use, simultaneous use of both pump and CGM, race, and insurance were all statistically significant predictors of HbA1c levels. As shown in Table 2, multiple linear regression demonstrated that higher ADI, Black racial identity, and Medicaid insurance were significantly associated with increased HbA1c levels, whereas insulin pump and CGM usage were significantly associated with lower HbA1c levels. The multiple linear regression model was significant with an adjusted R^2^ value of 0.206, and ANOVA analysis found F (11,1459) = 35.5, *p* < 0.001. (Figs. 2, 3).

**Table 2:** Multiple linear regression using mean HbA1c as output variable

	Unstandardized Coefficients		Standardized Coefficients			95% CI for Beta	
	B	Std. Error	Beta	t	p	Lower	Upper
**Age**	0.014	0.01	0.032	1.374	0.17	−0.006	0.034
**ADI**	0.006	0.002	0.078	2.997	0.003	0.002	0.01
**Male Sex**	0.134	0.089	0.035	1.507	0.132	−0.04	0.309
**Number of Encounters**	0.003	0.016	0.005	0.194	0.846	−0.028	0.034
**Race**							
Black/African American	1.386	0.142	0.248	9.781	< 0.001	1.108	1.664
Other	−0.328	0.303	−0.025	−1.08	0.28	−0.922	0.267
**Insurance**							
Medicaid	0.66	0.115	0.154	5.75	< 0.001	0.435	0.886
Other	0.102	0.176	0.014	0.581	0.561	−0.243	0.448
**Diabetes Technology**							
Combined Pump and CGM Use	−0.054	0.217	−0.014	−0.25	0.803	−0.481	0.372
Insulin Pump Use	−0.41	0.179	−0.108	2.289	0.022	−0.762	−0.059
CGM Use	−0.345	0.132	−0.091	−2.61	0.009	−0.604	−0.086

#### DKA

Unstandardized residuals for DKA did not demonstrate a normal distribution (Kolmogorov-Smirnov *p* < 0.001). Poisson regression demonstrated deviance Value/df of 1.433, a Pearson Chi-Square Value/df of 1.842, and AIC of 3272.02, suggestive of overdispersion and a poor model fit. Negative binomial regression demonstrated a deviance Value/df of 0.805, a Pearson Chi-Square Value/df of 1.036, and AIC of 2885.9. As shown in Table 3, the negative binomial regression demonstrated that age, HbA1c, number of encounters, Black/African American racial identity, and Medicaid insurance were associated with significantly increased rates of DKA (*p* < 0.001, *p* < 0.001, *p* < 0.001, *p* = 0.01, *p* = 0.001, respectively). Additionally, insulin pump use was associated with increased rates of DKA (*p* = 0.03). However, both combined CGM and insulin pump use and CGM use were not associated with lower rates of DKA (*p* = 0.06, *p* = 0.344, respectively).

**Table 3:** Negative binomial regression with DKA occurrences as outcome variable

					95% CI for exp (b)	
	B	Std. Error	Sig.	exp (b)	Lower	Upper
Age	0.050	0.011	< 0.001	1.051	1.028	1.075
National ADI	0.001	0.002	0.707	1.001	0.997	1.005
Male Sex	−0.012	0.093	0.897	0.988	0.823	1.187
Mean HbA1c	0.261	0.025	< 0.001	1.298	1.236	1.362
Number of Encounters	0.128	0.013	< 0.001	1.136	1.108	1.165
**Race**						
Other Race	0.314	0.302	0.299	1.369	0.757	2.476
Black/African American	0.336	0.131	0.010	1.400	1.082	1.810
**Insurance**						
Other Insurance	0.582	0.169	0.001	1.790	1.287	2.491
Medicaid Insurance	0.391	0.115	0.001	1.478	1.180	1.851
**Diabetes Technology**						
Combined Pump + CGM Use	−0.405	0.217	0.062	0.667	0.436	1.021
Pump Use	0.386	0.178	0.030	1.471	1.039	2.085
CGM Use	0.126	0.133	0.344	1.134	0.874	1.472

## Discussion

In this study, Black patients exhibited approximately 2% higher HbA1c and higher rates of Diabetic Ketoacidosis (DKA) compared to White patients, validating the impact of racial and socioeconomic disparities in glycemic control in youth with T1DM. HbA1c levels in this cohort were influenced by technology use, race, insurance, and higher social adversity as indicated by higher Area Deprivation Index (ADI) scores. While Black patients had higher rates of Medicaid use and higher ADI scores compared to White patients, the relationship between HbA1c levels and race remained independent of socioeconomic and insurance status. Interestingly, unlike findings from other studies, lower socioeconomic status and CGM use did not emerge as statistically significant predictors of DKA [20]. This data suggests that targeted interventions designed to improve HbA1c, such as the use of continuous glucose monitors in marginalized communities, could still positively influence DKA admission rates. Even though our study population was more disadvantaged than the national average, both this study and others indicate that the factors contributing to racial disparities in glycemic control among youth with T1DM go beyond ADI, insurance type and technology use. Other studies have suggested that factors influenced by institutional racism may be involved [6, 14].

Despite the demonstrated benefits of diabetes technology in improving patient outcomes for T1DM, many patients in the United States and around the world still lack access to these technologies. Prescription and usage of diabetes technology are influenced by various factors, including availability, affordability, differences in coverage among payor systems in different states, preferences of patients and their families, and their trust in the healthcare system, among others [21, 22]. Process barriers may also impact a patient or family’s willingness or ability to access diabetes technology. In our clinical practice, CGMs are typically initiated at diagnosis after discussion of insurance coverage and out-of-pocket costs with families. Pump therapy can be initiated as early as one month after diagnosis and requires participation of patients and/or caregivers in a brief pump course and knowledge assessment.

This study was conducted at a time when insurance coverage for diabetes technologies was limited in our state. This may explain why only 56% of the patients in this study were using a CGM and 45% had an insulin pump. This allowed us to ascertain the effects of limited technology access in underserved communities as it occurs in many regions around the world. Thus, as we continue to advocate for improved access and coverage of diabetes technology for all patients, this study suggests that the impact of such access may be even more significant for the most vulnerable patients.

This and other studies indicate that racial and socioeconomic disparities in technology usage exist among youth with T1DM [6–14]. However, further research is needed to effectively evaluate the trends and motivations behind diabetes technology prescription from all involved parties and to better understand the factors that contribute to the successful use of such technology. Prior studies suggest that provider bias may lead to racial disparities in diabetes technology use, with Black patients facing lower rates of device initiation and higher rates of device discontinuation [17, 23]. However, children from racial and socially disadvantaged backgrounds remain significantly under-represented in clinical trials for FDA-approved modern diabetes technologies. This critical gap underscores the urgent need to study diverse populations of children with T1DM to better understand how these technologies impact these underserved patients. Our cohort included youth from various racial and socioeconomic backgrounds, reflecting the makeup of the 2022 US population, and a the percentage of patients identifying as Black was slightly higher than typically reported among T1DM populations [24]. Therefore, this study provides a valuable opportunity to examine the relationship between social vulnerability and diabetes outcomes, as well as how the use of diabetes technology, such as CGM or Continuous subcutaneous insulin infusion via insulin pump (CSII) affects diabetes outcomes across different populations.

CSII offers several advantages over multiple daily injections including a reduction in the disease burden of patients with T1DM and is associated with better glycemic control in numerous studies [25, 26]. While insulin pump usage has been rising substantially among young individuals with T1DM, adoption rates for diabetes technology in many vulnerable populations remain notably lower than the national average [14]. This study indicated lower usage of CGM and pumps among black patients, which may be related to insurance coverage at the time of the study. Although we acknowledge that improvements in glycemic control may be driven by more individualized insulin dosing, prompt pump adaptation to temporary changes in insulin sensitivity, and increased user satisfaction [27] provided by diabetes technology, our findings showed that the use of insulin pumps or CGMs alone is associated with a lower HbA1c levels, even after accounting for other variables. Furthermore, these data suggest that while insulin pump or CGM use is associated with lower HbA1c levels in all patients with T1DM, this difference was especially notable in patients with higher ADI scores and in Black patients.

Prior studies have noted both increases and decreases in rates of DKA in patients with insulin pump use [28, 29]. This study found an association between insulin pump use and increased DKA occurrences. Insulin pump use has a theoretical higher risk of DKA as CSII exclusively uses short acting insulin, which if disrupted and unrecognized could lead to poorer short term glycemic control and DKA. Additionally, insulin pump therapy has been shown to be more expensive to patients and their families than insulin injection, which may lead to increased difficulty adhering to insulin therapy [30]. Rates of DKA in individuals with insulin pump use in existing diabetes literature may vary due to availability of CSII closed loop systems. During our study period, legislation may have influenced availability of these systems. Our study population was significantly more disadvantaged than the national average and may be exposed to additional social determinants of health that are not able to be encumbered by our study design, leading to increased rates of DKA admissions associated with insulin pump use.

Although this is not a clinical trial and we are limited to draw definitive conclusions regarding the direct impact of pump or CGM use on glycemic control, this study suggests that the use of DM technology might mitigate the effects of social vulnerability on glycemic outcomes. This study also indicates that pump usage alone could eliminate racial disparities in improving A1c levels among youth with T1DM. While we recognize the clinical advantages of automated insulin delivery systems, we also note that access to these systems is limited both globally around the world and within the US. Consequently, our analysis focused on the independent use of pumps and CGMs rather than their combined use. Additionally, we recognize that our study methodology prevented an assessment of impact of CGM metrics such as time in range and wear time; however, we used HbA1c as an appropriate and standard method for assessing glycemic control because it reflects the average blood glucose over time and predicts diabetes complications. We expect a wide variability in how patients approach technology among the patients included in this populational study; but our results still demonstrate the positive association between technology use and glycemic control across the socioeconomic spectrum. Despite limitations on generalizability to areas with no technology delivery gaps, these data emphasize the need for national and global advocacy to reduce disparities in diabetes care delivery, particularly within payer systems that impose high eligibility requirements for access to diabetes technology. Moreover, this data challenges prescribing practices that require patients to achieve glycemic control benchmarks without technological assistance prior to gaining access to such technology. Lastly, we recognize that due to the study’s time frame, we are unable to determine the potential influence of the COVID-19 pandemic on glycemic control and the uptake of diabetes technology in this population.

## Conclusions

This study highlights the disparities in the use of diabetes technology and glycemic control among disadvantaged and socially vulnerable populations. Although hybrid closed-loop systems are becoming more commonly used, many patients worldwide still lack access to this technology. This study highlights that both insulin pump and CGM use alone can effectively lower HbA1c levels across all groups, with greater improvements observed in patients of lower socioeconomic background. Therefore, further research is needed to better understand the factors contributing to disparities in diabetes technology usage. This understanding can lead to new interventions aimed to mitigate and address the racial and socioeconomic inequalities faced by youth with type 1 diabetes.

## Declarations

### Data availability

Datasets analyzed during this study are not publicly available for privacy reasons. Requests for de-identified data can be made by contacting the corresponding author (AMA).

## References

[1] Laffel LM, Kanapka LG, Beck RW, et al. Effect of continuous glucose monitoring on glycemic control in adolescents and young adults with type 1 diabetes: a randomized clinical trial. JAMA. 2020;323(23):2388–96. 10.1001/jama.2020.6940. 32543683 10.1001/jama.2020.6940 PMC7298603

[2] Wong JC, Dolan LM, Yang TT, Hood KK. Insulin pump use and glycemic control in adolescents with type 1 diabetes: predictors of change in method of insulin delivery across two years. Pediatr Diabetes. 2015;16(8):592–99. 10.1111/pedi.12221. 25387433 10.1111/pedi.12221 PMC4458222

[3] Pańkowska E, Błazik M, Dziechciarz P, Szypowska A, Szajewska H. Continuous subcutaneous insulin infusion vs. multiple daily injections in children with type 1 diabetes: a systematic review and meta-analysis of randomized control trials. Pediatr Diabetes. 2009;10(1):52–58. 10.1111/j.1399-5448.2008.00440.x. 18761648 10.1111/j.1399-5448.2008.00440.x

[4] van Beers CAJ, Jh D. Continuous glucose monitoring: impact on hypoglycemia. J Diabetes Sci Technol. 2016;10(6):1251–58. 10.1177/1932296816653411. 27257169 10.1177/1932296816653411 PMC5094331

[5] van Beers CAJ, Jh D, Kleijer SJ, et al. Continuous glucose monitoring for patients with type 1 diabetes and impaired awareness of hypoglycaemia (IN CONTROL): a randomised, open-label, crossover trial. Lancet Diabetes Endocrinol. 2016;4(11):893–902. 10.1016/S2213-8587(16)30193-0. 27641781 10.1016/S2213-8587(16)30193-0

[6] Agarwal S, Schechter C, Gonzalez J, Long JA. Racial-ethnic disparities in diabetes technology use among young adults with type 1 diabetes. Diabetes Technol Ther. 2021;23(4):306–13. 10.1089/dia.2020.0338. 33155826 10.1089/dia.2020.0338 PMC7994432

[7] Lee MY, Tanenbaum ML, Maahs DM, Prahalad P. Overcoming barriers to diabetes technology in youth with type 1 diabetes and public insurance: cases and call to action. Case Rep Endocrinol. 2022, 2022;9911736. 10.1155/2022/9911736. 10.1155/2022/9911736 PMC8904094 35273814

[8] Allison K, Patel D, Kaur R. Assessing multiple factors affecting minority participation in clinical trials: development of the clinical trials participation barriers Survey. Cureus. 2022;14(4):e24424. 10.7759/cureus.24424. 35637812 10.7759/cureus.24424 PMC9127181

[9] Tremblay ES, Bernique A, Garvey K, Astley CM. A retrospective cohort study of racial/ethnic and socioeconomic disparities in initiation and meaningful use of Continuous glucose monitoring among youth with type 1 diabetes. J Diabetes Sci Technol. Published online July 3, 2023: 19322968231183985. 10.1177/19322968231183985. 10.1177/19322968231183985 PMC11531052 37394962

[10] S M, O E, N N, et al. Inequities in health outcomes in children and adults with type 1 diabetes: data from the T1D exchange quality improvement collaborative. Clin Diabetes: A Publ Of The Am Diabetes Assoc. 2021;39(3). 10.2337/cd21-0028. 10.2337/cd21-0028 PMC8329009 34421203

[11] Baboun D, Solano N, Del Toro V, Alvarez-Salvat R, Granados A, Carrillo-Iregui A. Technology use and clinical outcomes in a racial-ethnic minority cohort of children and adolescents with type 1 diabetes. J Pediatr Endocrinol Metab. 2023;36(12):1128–32. 10.1515/jpem-2023-0334. 37852007 10.1515/jpem-2023-0334

[12] McKee AM, Al-Hammadi N, Hinyard LJ. Disparities in utilization and outcomes with continuous subcutaneous insulin infusion in young adults with type 1 diabetes. Endocr Pract. 2021;27(8):769–75. 10.1016/j.eprac.2021.05.001. 33991655 10.1016/j.eprac.2021.05.001

[13] Ebekozien O, Mungmode A, Sanchez J, et al. Longitudinal trends in glycemic outcomes and technology use for over 48,000 people with type 1 diabetes (2016-2022) from the T1D exchange quality improvement collaborative. Diabetes Technol Ther. 2023;25(11):765–73. 10.1089/dia.2023.0320. 37768677 10.1089/dia.2023.0320

[14] Fantasia KL, Wirunsawanya K, Lee C, Rizo I. Racial disparities in diabetes technology use and outcomes in type 1 diabetes in a safety-net hospital. J Diabetes Sci Technol. 2021;15(5):1010–17. 10.1177/1932296821995810. 33719610 10.1177/1932296821995810 PMC8442173

[15] Lipman TH, Smith JA, Patil O, Willi SM, Hawkes CP. Racial disparities in treatment and outcomes of children with type 1 diabetes. Pediatr Diabetes. 2021;22(2):241–48. 10.1111/pedi.13139. 33871154 10.1111/pedi.13139

[16] Sm O, Sh G. Social determinants of health and structural inequities-root causes of diabetes disparities. Diabetes Care. 2021;44(1). 10.2337/dci20-0060. 10.2337/dci20-0060 33571949

[17] Addala A, Hanes S, Naranjo D, Maahs DM, Hood KK. Provider implicit bias impacts pediatric type 1 diabetes technology recommendations in the United States: findings from the gatekeeper study. J Diabetes Sci Technol. 2021;15(5):1027–33. 10.1177/19322968211006476. 33858206 10.1177/19322968211006476 PMC8442183

[18] Kind AJH, Buckingham WR. Making Neighborhood-disadvantage metrics accessible - the Neighborhood Atlas. N Engl J Med. 2018;378(26):2456–58. 10.1056/NEJMp1802313. 29949490 10.1056/NEJMp1802313 PMC6051533

[19] VanderPlas J, Granger B, Heer J, et al. Altair: interactive statistical visualizations for python. JOSS. 2018;3(32):1057. 10.21105/joss.01057.

[20] Wersäll JH, Ekelund J, Åkesson K, et al. Relative poverty is associated with increased risk of diabetic ketoacidosis at onset of type 1 diabetes in children: a Swedish national population-based study in 2014–2019. Diabet Med. 2024;41(7):e15283. 10.1111/dme.15283. 38213059 10.1111/dme.15283

[21] Papadakis JL, Anderson LM, Garza K, et al. Psychosocial aspects of diabetes technology use. Endocrinol Metab Clinics of North Am. 2020;49(1):127–41. 10.1016/j.ecl.2019.10.004. 10.1016/j.ecl.2019.10.004 31980113

[22] de Vries Sag, Bak JCG, Verheugt CL, et al. Healthcare expenditure and technology use in pediatric diabetes care. BMC Endocr Disord. 2023;23(1):72. 10.1186/s12902-023-01316-3. 37029362 10.1186/s12902-023-01316-3 PMC10080182

[23] Sora ND, Shashpal F, Bond EA, Jenkins AJ. Insulin pumps: review of technological advancement in diabetes management. Am J Med Sci. 2019;358(5):326–31. 10.1016/j.amjms.2019.08.008. 31655714 10.1016/j.amjms.2019.08.008

[24] Everett EM, Wright D, Williams A, et al. A longitudinal view of disparities in insulin pump use among youth with type 1 diabetes: the SEARCH for diabetes in youth study. Diabetes Technol & Ther. 2023;25(2):131–39. 10.1089/dia.2022.0340. 36475821 10.1089/dia.2022.0340 PMC9894603

[25] M AB, Nk S, As B, R E. Insulin pumps in children - a systematic review. World J Clin Pediatrics. 2022;11(6). 10.5409/wjcp.v11.i6.463. 10.5409/wjcp.v11.i6.463 PMC9685680 36439904

[26] REPOSE Study Group. Relative effectiveness of insulin pump treatment over multiple daily injections and structured education during flexible intensive insulin treatment for type 1 diabetes: cluster randomised trial (REPOSE). BMJ. 2017;356:j1285. 10.1136/bmj.j1285. 28360027 10.1136/bmj.j1285 PMC6276869

[27] Nimri R, Nir J, Phillip M. Insulin pump therapy. Am J Ther. 2020;27(1):e30–41. 10.1097/MJT.0000000000001097. 31833871 10.1097/MJT.0000000000001097

[28] Blackman SM, Raghinaru D, Adi S, et al. Insulin pump use in young children in the T1D Exchange clinic registry is associated with lower hemoglobin A1c levels than injection therapy. Pediatr Diabetes. 2014;15(8):564–72. 10.1111/pedi.12121. 10.1111/pedi.12121 24494980

[29] Karges B, Schwandt A, Heidtmann B, et al. Association of insulin pump therapy vs insulin injection therapy with severe hypoglycemia, ketoacidosis, and glycemic control among children, adolescents, and young adults with type 1 diabetes. JAMA. 2017;318(14):1358–66. 10.1001/jama.2017.13994. 29049584 10.1001/jama.2017.13994 PMC5818842

[30] Toresson Grip E, Svensson AM, Miftaraj M, et al. Real-world costs of continuous insulin pump therapy and multiple daily injections for type 1 diabetes: a population-based and propensity-matched cohort from the Swedish national diabetes register. Diabetes Care. 2019;42(4):545–52. 10.2337/dc18-1850. 30705062 10.2337/dc18-1850

